# Integrating psychological resilience into community health strategies: addressing stigma-induced social alienation in postoperative colorectal cancer patients

**DOI:** 10.3389/fpubh.2025.1618599

**Published:** 2025-07-16

**Authors:** Xin Guo, Yuqin Wang, Qi Han, Rui Zhang, Shuyu Liu, Yuenan Huang, Yan Zhang, Botang Guo

**Affiliations:** ^1^Department of General Surgery, The First Affiliated Hospital of Harbin Medical University, Harbin, Heilongjiang, China; ^2^Department of Psychological Sleep, The Psychological Health Center of Harbin First Specialized Hospital, Harbin, Heilongjiang, China; ^3^Department of General Surgery, The Second Affiliated Hospital of Harbin Medical University, Harbin, Heilongjiang, China; ^4^Department of Internal Medicine, Shenzhen Guangming District Maternal and Child Health Hospital, Shenzhen, Guangdong, China; ^5^Department of General Practice, The Affiliated Luohu Hospital of Shenzhen University Medical School, Shenzhen, Guangdong, China

**Keywords:** colorectal cancer, stigma, social alienation, psychological resilience, community health, postoperative care

## Abstract

**Background:**

Colorectal cancer (CRC) is a leading cause of mortality globally. While survival rates have improved, postoperative patients face psychosocial challenges such as social alienation and stigma, which affect their recovery. Psychological resilience may serve as a protective factor, but its role in mediating the effects of stigma on social alienation in CRC patients is not well understood.

**Objective:**

This study aims to examine the mediating role of psychological resilience in the relationship between perceived stigma and social alienation among postoperative colorectal cancer patients.

**Method:**

A cross-sectional study was conducted with 382 postoperative CRC patients from three tertiary hospitals in Harbin and Shenzhen, China, between January 2023 and December 2024. Participants completed self-report measures of perceived stigma, psychological resilience, and social alienation. Data were analyzed using hierarchical regression and bootstrapping to test the mediation model.

**Result:**

Perceived stigma was positively correlated with social alienation and negatively with psychological resilience. Psychological resilience partially mediated the relationship between stigma and social alienation, explaining 30.8% of the total effect. Significant differences in social alienation were found based on gender, age, and stoma status.

**Conclusion:**

Psychological resilience plays a crucial role in reducing social alienation in postoperative CRC patients. Interventions focused on enhancing resilience could help mitigate stigma and improve social reintegration. Community-based resilience programs are recommended for supporting CRC survivors.

## 1 Introduction

Colorectal cancer (CRC) is one of the most commonly diagnosed malignancies worldwide, ranking third in incidence and second in mortality (1). With advancements in screening and surgical techniques, early detection and treatment of CRC have become more accessible. These improvements have led to higher survival rates and an increasing number of long-term survivors (2). However, patients still face significant postoperative challenges that go beyond physical recovery (3). These include emotional distress, altered body image, and difficulty reintegrating into social life. Together, these factors may hinder rehabilitation and reduce quality of life (4).

As health systems shift from acute, hospital-based care to chronic disease management, community-based care has gained importance (5, 6). Community nursing not only offers ongoing medical follow-up but also delivers psychosocial support. It helps facilitate patients' return to social life and promotes adherence to healthy behaviors (7). Community-based health education—through peer support, workshops, or digital platforms—can enhance self-efficacy and reduce disease-related misconceptions (8). It also helps foster a more inclusive social environment. For CRC survivors recovering at home, such community-level interventions are essential for sustained recovery and social reintegration (9).

Among the major psychosocial challenges confronting CRC survivors is social alienation—a condition marked by reduced social participation, feelings of isolation, and perceived disconnection from others (10). This problem is especially pronounced among postoperative patients who experience physical aftereffects such as a stoma, bowel dysfunction, or chronic fatigue. These conditions can lead to embarrassment and cause patients to avoid public or social interaction (11). Mounting evidence suggests that social alienation reduces quality of life and raises the risk of mental health problems. It may also negatively affect long-term health outcomes (12).

One key psychological factor associated with social alienation is perceived stigma. In illness contexts, stigma involves feelings of shame, inferiority, or fear of social judgment due to one's health status or visible symptoms (13). CRC survivors may feel stigmatized because of altered bowel habits, visible colostomy bags, or fear of misunderstanding by others (14). When this stigma is internalized, it often causes social withdrawal and deepens the sense of alienation.

However, not all patients are equally affected. Stress and coping theory proposes that psychological reactions to illness-related stressors—such as stigma—are shaped by coping resources like resilience (15, 16). Psychological resilience is defined as the capacity to adapt positively to stress and adversity (17). It may buffer the harmful effects of stigma. Resilient individuals are more likely to stay emotionally stable, seek support, and maintain social engagement despite illness (18). Importantly, resilience is a changeable trait. It can be strengthened through targeted interventions. Community-based education and psychosocial support offer effective ways to build resilience in CRC survivors (19).

Previous studies in breast cancer (20), HIV (21), and lung cancer (22) populations have identified the mediating role of resilience in the stigma-psychological distress relationship. However, little is known about this mechanism in the context of CRC, particularly during the early stages of postoperative recovery when stigma and social vulnerability are most pronounced.

**Figure 1:**
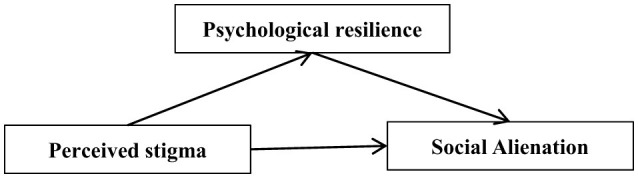
Research conception model.

Therefore, the present study seeks to investigate the mediating role of psychological resilience in the relationship between perceived stigma and Social Alienation in postoperative colorectal cancer patients (Figure 1). Based on the Stress and Coping Theory, propose the following hypotheses:

H1: Perceived stigma is positively associated with social alienation;
H2: Perceived stigma is negatively associated with psychological resilience;
H3: Psychological resilience is negatively associated with social alienation;
H4: Psychological resilience mediates the relationship between perceived stigma and social alienation.

This study aims to clarify the psychological mechanisms that hinder social reintegration among CRC survivors by testing the proposed hypotheses. It also seeks to provide empirical support for resilience-based interventions in community nursing and educational programs. These efforts may help promote more holistic and patient-centered colorectal cancer care.

## 2 Methods

### 2.1 Participants

This cross-sectional study was conducted between January 2023 and December 2024 at three tertiary general hospitals located in Harbin and Shenzhen, China. A convenience sampling method was employed to recruit participants from the general surgery departments of these hospitals. Eligible participants were identified by attending physicians and invited to participate if they met the following criteria: (1) aged 18 years or older; (2) histologically confirmed diagnosis of CRC and had undergone radical surgery; (3) clear awareness of their disease status; and (4) the ability to read and complete questionnaires independently or with assistance. Patients were excluded if they: (1) had cognitive impairment or major psychiatric illness; (2) had metastatic disease or severe organ dysfunction; or (3) were currently enrolled in psychosocial intervention programs. A total of 382 participants were enrolled. All participants provided written informed consent prior to participation. The study was approved by the Ethics Committee of the First Affiliated Hospital of Harbin Medical University (2024IIT179).

### 2.2 Instruments

#### 2.2.1 Demographic questionnaire

Demographic and clinical characteristics, including gender, age, marital status, education, monthly income, stoma status, chemotherapy history, and time post surgery, were collected through structured questionnaires and medical record review.

#### 2.2.2 Social alienation

Social alienation was assessed using a 17-item instrument derived from validated research on cancer patients, with each item rated on a five-point Likert scale (1 = strongly disagree to 5 = strongly agree) (23). The total score ranges from 17 to 85, where higher scores represent greater perceived social alienation. The Cronbach's α in the present sample was 0.84.

#### 2.2.3 Perceived stigma

Perceived Stigma was measured using Social Impact Scale (24). The scale consists of 24 items, divided into four dimensions: social isolation (seven items), economic discrimination (three items), social exclusion (nine items), and internal shame (five items). The items are scored using the Likert four-point scoring system, where 4 = strongly agree, 3 = agree, 2 = disagree, and 1 = strongly disagree. The total score of the scale is the sum of the scores of each item, ranging from 24 to 96 points. The higher the score, the higher the perceived sense of shame. The Cronbach's α for the scale in this study was 0.86.

#### 2.2.4 Resilience scale specific to cancer

This scale is the only scale developed to evaluate the psychological resilience of cancer patients (25). It includes 25 items divided into five dimensions: nonspecific resilience components (Items 1–6), disease benefits (Items 7–11), support and response (Items 12–16), hope for the future (Items 17–21), and meaning of existence (Items 22–25). The scale is scored using a five-point Likert scale, and the total score is 25–125 points. The higher the score is, the better the resilience. The Cronbach's α coefficient is 0.85, and the structural validity is 0.901, which indicates good reliability and validity.

### 2.3 Quality control

All investigators and data collectors received standardized training before data collection. Questionnaires were completed in a quiet room prior to hospital discharge or during outpatient follow-up. Participants with limited literacy were assisted by trained researchers to ensure consistency and data validity. Double data entry and consistency verification were performed using EpiData 3.1 to minimize input errors.

### 2.4 Statistical analysis

Data analysis was performed using SPSS version 26.0. Descriptive statistics were computed for all variables. Independent sample *t*-tests or ANOVA were used for univariate analyses to examine differences in social alienation scores across demographic groups. Pearson's correlation coefficients were calculated to assess associations between stigma, resilience, and social alienation. To test the hypothesized mediation model, we conducted a series of hierarchical regression analyses following the procedures proposed by Baron and Kenny. Bootstrapping with 5,000 resamples was used to estimate the indirect effects and corresponding 95% confidence intervals. Mediation was confirmed if the indirect effect was significant and the confidence interval excluded zero. The proportion of mediation was calculated as the ratio of the indirect effect to the total effect. A two-tailed *P*-value < 0.05 was considered statistically significant.

## 3 Results

### 3.1 Demographic characteristics

382 postoperative colorectal cancer patients were included. Most participants were aged 61–70 years (47.38%), male (61.52%), and married (80.89%). Regarding education, 69.63% had completed high school, and 8.38% held a bachelor's degree or above. Monthly income was ≤ 5,000 yuan in 81.15% of patients. Stoma formation was reported in 29.32% of cases, and 58.64% had received chemotherapy. Time since surgery was >12 months in 30.63% of patients, 7–12 months in 40.05%, and ≤ 6 months in 29.32%. Independent *t*-tests and ANOVAs revealed significant differences in social alienation scores across several demographic subgroups. Females reported significantly higher social alienation scores than males (*t* = −4.791, *P* < 0.001). Participants younger than 60 years showed significantly higher levels of alienation compared to those aged 60–70 or above 70 (*F* = 12.561, *P* < 0.001). Patients with a stoma also reported higher alienation (*t* = −7.832, *P* < 0.001). Regarding postoperative time, patients more than 12 months post surgery had significantly higher alienation scores compared to those earlier in recovery (*F* = 24.796, *P* < 0.001). Marital status and education level approached significance, while chemotherapy status did not show a significant association with social alienation. Details were presented in Table 1.

**Table 1:** Demographic differences in students social alienation (*N* = 1,110).

**Variable**	***n* (%)**	**Social alienation, mean ±SD**	***F/t***	***P*-value**
**Age (year)**				
≤ 60	62 (16.23)	83.13 ± 2.91	12.561	< 0.001
61–70	181 (47.38)	80.02 ± 4.91		
>70	139 (36.39)	79.81 ± 4.83		
**Gender**				
Male	235 (61.52)	79.59 ± 4.94	−4.791	< 0.001
Female	147 (38.48)	81.82 ± 4.09		
**Marital status**				
Married	309 (80.89)	80.74 ± 4.69	3.242	0.040
Unmarried	49 (12.83)	79.06 ± 4.87		
Divorced	24 (6.28)	79.46 ± 4.95		
**Education level**				
Junior and below	84 (21.99)	81.36 ± 4.12	2.650	0.072
High school	266 (69.63)	80.30 ± 4.81		
Bachelor or above	32 (8.38)	79.28 ± 5.53		
**Monthly income (yuan)**				
≤ 3,000	157 (41.10)	80.43 ± 5.09	1.804	0.166
3,001–5,000	153 (40.05)	80.05 ± 4.57		
>5,000	72 (18.85)	81.33 ± 4.31		
**Stoma**				
No	270 (70.68)	79.31 ± 4.93	61.357	< 0.001
Yes	112 (29.32)	83.20 ± 2.83		
**Chemotherapy**				
No	158 (41.36)	80.68 ± 4.74	0.663	0.416
Yes	224 (58.64)	80.28 ± 4.77		
**Post surgery (month)**				
≤ 6	112 (29.32)	79.54 ± 4.45	24.796	< 0.001
7–12	153 (40.05)	79.25 ± 5.18		
>12	117 (30.63)	82.87 ± 3.41		

### 3.2 Common method deviation test

To assess the potential for common method variance, Harman's single-factor test was employed. An exploratory factor analysis of all items across the three psychological scales yielded 27 factors with eigenvalues >1. The first factor accounted for 3.195% of the total variance, which is below the critical threshold of 40%, indicating that common method bias was not a significant concern in this study.

### 3.3 Correlation analysis

The mean scores for perceived stigma, psychological resilience, and social alienation were (66.89 ± 2.80), (48.82 ± 4.12), and (80.45 ± 4.75), respectively (Table 2). Pearson correlation analysis revealed that perceived stigma was positively correlated with social alienation (*r* = 0.282, *P* < 0.01) and negatively correlated with psychological resilience (*r* = −0.457, *P* < 0.01). Additionally, psychological resilience was negatively correlated with social alienation (*r* = −0.279, *P* < 0.01).

**Table 2:** Description statistics and correlation analysis of each variable.

**Variables**	**Mean ±SD**	**1**	**2**	**3**
1. Social alienation	80.45 ± 4.75	1		
2. Stigma	66.89 ± 2.80	0.282^**^	1	
3. Psychological resilience	48.82 ± 4.12	−0.279^**^	−0.457^**^	1

^**^P < 0.01.

### 3.4 Mediation analysis

**Figure 2:**
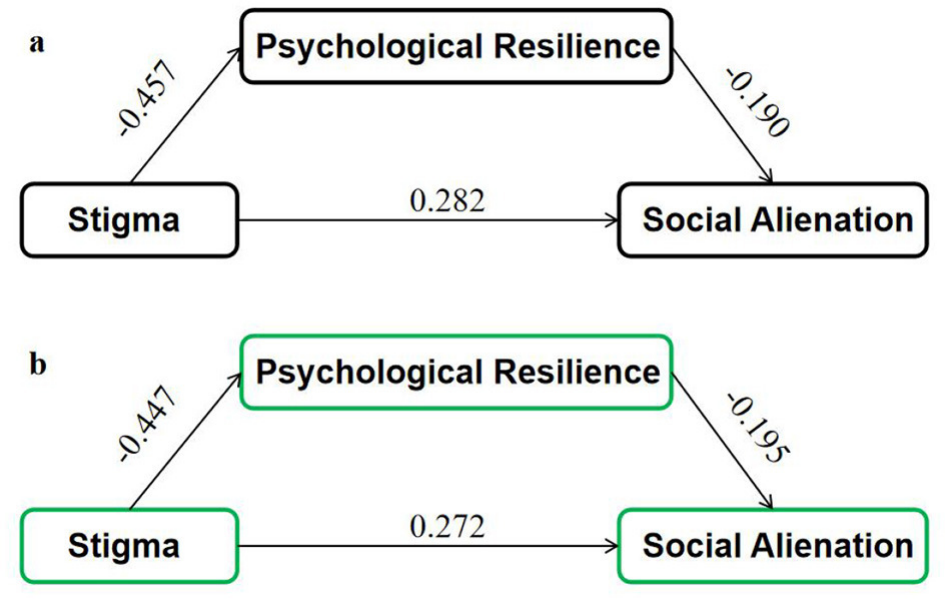
Mediation models of the effect of stigma on social alienation via psychological resilience. **(a)** Unadjusted model, **(b)** adjusted model controlling for gender, age, stoma status, and postoperative time.

Hierarchical regression analyses based on Baron and Kenny's approach confirmed the mediating role of psychological resilience in the relationship between stigma and social alienation. In the first step, stigma significantly predicted social alienation (*B* = 0.276, *P* < 0.001). In the second step, stigma significantly predicted psychological resilience (*B* = −0.388, *P* < 0.001). In the third step, when both stigma and psychological resilience were included as predictors, stigma (*B* = 0.191, *P* < 0.001) and psychological resilience (*B* = −0.219, *P* < 0.001) significantly predicted social alienation (Table 3). Bootstrapping analysis (5,000 samples) showed that the indirect effect was 0.085 [95% CI: (0.038, 0.139)], while the direct effect was 0.191 [95% CI: (0.086, 0.296)], accounting for 30.8% of the total effect, confirming a statistically significant partial mediation (Figure 2).

**Table 3:** Summary of hierarchical regression analyses predicting social alienation.

**Regression equation**		**Overall fit coefficient**			**Regression coefficient**			
**Outcome variables**	**Predictor variables**	***R***	***R** ^2^*	***F***	β	***B***	**SE**	***t***
Social alienation	Stigma	0.282	0.079	32.787^**^	0.282	0.276	0.048	5.726^**^
Psychological resilience	Stigma	0.457	0.209	100.479^**^	−0.457	−0.388	0.039	−10.024^**^
Social alienation	Stigma	0.328	0.108	22.905^**^	0.195	0.191	0.053	3.577^**^
	Psychological resilience				−0.190	−0.219	0.063	−3.474^**^

^**^P < 0.01.

### 3.5 Sensitivity analysis

To ensure robustness, a sensitivity analysis was conducted by including gender, age group, stoma status, and postoperative time as covariates in the mediation model. The mediating effect of psychological resilience remained significant [indirect effect = 0.082, 95% CI: (0.035, 0.128)], with a mediation proportion of 29.6%. All key demographic covariates remained significant predictors of social alienation, indicating that the mediation model was stable across different subgroups. Results were presented in Table 4 and Figure 2.

**Table 4:** Summary of hierarchical regression analyses predicting social alienation.

**Regression equation**		**Overall fit coefficient**			**Regression coefficient**			
**Outcome variables**	**Predictor variables**	***R***	***R** ^2^*	***F***	β	***B***	**SE**	***t***
Social alienation	Stigma	0.646	0.417	44.673^**^	0.272	0.266	0.039	6.855^**^
	Gender				0.272	2.656	0.386	6.882^**^
	Age				−0.235	−1.598	0.271	−5.908^**^
	Marital status				−0.067	−0.567	0.335	−1.691
	Stoma				0.379	3.957	0.413	9.577^**^
	Post surgery time				0.298	1.828	0.243	7.522^**^
Psychological resilience	Stigma	0.476	0.226	18.273^**^	−0.447	−0.380	0.039	−9.801^**^
	Gender				0.020	0.165	0.386	0.429
	Age				0.023	0.136	0.270	0.503
	Marital status				0.077	0.563	0.335	1.683
	Stoma				−0.046	−0.412	0.413	−0.998
	Postoperative time				0.087	0.462	0.243	1.904
Social alienation	Stigma	0.668	0.446	43.042^**^	0.185	0.181	0.042	4.257^**^
	Psychological resilience				−0.195	−0.225	0.051	−4.451^**^
	Gender				0.276	2.694	0.377	7.149^**^
	Age				−0.230	−1.567	0.264	−5.936^**^
	Marital status				−0.052	−0.440	0.328	−1.341
	Stoma				0.371	3.864	0.404	9.572^**^
	Postoperative time				0.315	1.932	0.238	8.107^**^

^**^P < 0.01.

## 4 Discussion

This study investigated the relationships among perceived stigma, psychological resilience, and social alienation in postoperative CRC patients. The findings revealed that social alienation levels were notably higher among patients who were younger ( ≤ 60 years), female, had undergone stoma formation, or were more than 12 months post surgery. Perceived stigma was positively correlated with social alienation and negatively correlated with psychological resilience. Furthermore, psychological resilience was found to partially mediate the relationship between perceived stigma and social alienation, accounting for ~30.8% of the total effect. These results underscore the significant role of psychological resilience in mitigating the adverse impact of stigma on social alienation among CRC patients.

The observed positive association between perceived stigma and social alienation is consistent with previous findings that stigma contributes to social withdrawal and isolation among cancer patients (26). For example, Wang et al. (27) reported that higher levels of perceived stigma were linked to greater social dysfunction in CRC patients with a stoma. Similarly, Wu et al. (28) found that stigma impaired social connectedness among stroke patients, resulting in increased feelings of alienation.

The mediating role of psychological resilience identified in this study is consistent with prior research. For instance, Ben Salah et al. (29) found that resilience mediated the relationship between sleep quality and social isolation during the COVID-19 pandemic. Similarly, a Chinese study by Wu et al. (30) showed that family resilience significantly influenced social isolation in stroke patients. These findings collectively indicate that psychological resilience acts as a protective factor against the adverse impact of stigma on social integration. Thus, interventions that strengthen resilience may help reduce social alienation in CRC patients.

The observed relationships among perceived stigma, psychological resilience, and social alienation in postoperative CRC patients can be effectively interpreted through Lazarus and Folkman's Stress and Coping Theory (31, 32). This theory suggests that psychological responses to stressors are shaped by individuals' cognitive appraisals and available coping resources. In this framework, perceived stigma acts as a psychosocial stressor, while psychological resilience serves as a coping mechanism that can buffer its negative effects (33, 34). Among CRC patients, stigma often stems from concerns about body image, bowel dysfunction, and the presence of a stoma—factors that may lead to shame and social withdrawal (35, 36). Such negative self-perceptions can intensify psychological stress and hinder reintegration into social life. Psychological resilience, defined as the ability to adapt positively in the face of adversity, helps individuals reframe these challenges, maintain emotional stability, and adopt active coping strategies. Higher resilience has been linked to improved social functioning and lower levels of isolation in cancer survivors (37). These findings highlight the protective role of resilience in mitigating the impact of stigma on social engagement. Moreover, resilience is dynamic and modifiable, meaning it can be strengthened through targeted interventions (38). Programs such as cognitive-behavioral therapy, peer support groups, and community-based initiatives have shown promise in enhancing resilience among cancer patients (39).

By strengthening these coping resources, such interventions may help alleviate perceived stigma and social alienation, thereby enhancing the quality of life among postoperative CRC patients. This study highlights the potential of community health education in improving the psychosocial wellbeing of postoperative CRC patients by addressing key factors such as perceived stigma and psychological resilience. Based on the Stress and Coping Theory, interventions that reduce stigma and enhance resilience can significantly mitigate social alienation and improve patient outcomes. To translate these findings into practice, operational strategies must be defined. A feasible community-based resilience program could involve collaboration among several key actors. Community health workers, including nurses and public health educators, can take the lead in delivering psychoeducation, organizing peer support groups, and conducting cognitive-behavioral training workshops tailored to the CRC population. These programs should be culturally adapted and regularly held in community health centers or through digital platforms to ensure broad accessibility. Hospitals can serve as referral hubs, identifying high-risk patients during postoperative follow-ups and connecting them to community programs. Social workers and psychologists may be mobilized through local health bureaus to provide individual or group interventions, especially for patients with high stigma levels. Additionally, digital health tools such as telehealth platforms and mobile apps can facilitate communication between patients and providers, enabling continuous psychosocial monitoring and resilience coaching. Policymakers should support the integration of these community services into routine cancer survivorship care, ensuring sustainable funding and standardized guidelines. By fostering hospital-community linkages and leveraging multidisciplinary teams, these operational models can effectively reduce social alienation and promote long-term recovery in CRC survivors.

## 5 Strengths and limitations

This study provides novel insights into the psychosocial dynamics of postoperative CRC patients by investigating the mediating role of psychological resilience in the relationship between perceived stigma and social alienation. The multicenter sampling from three tertiary hospitals in Harbin and Shenzhen improves the generalizability of the findings across geographically and socioeconomically diverse populations. Additionally, the use of validated instruments for all key constructs enhances the study's methodological rigor and construct validity. However, several limitations should be acknowledged. First, the cross-sectional design limits causal inferences, and the directionality of the observed associations cannot be confirmed. Second, the use of self-reported questionnaires may be subject to recall or social desirability biases. Third, the study did not assess important covariates such as patients' perceived social support, pre-existing mental health conditions, or concurrent life stressors, which could confound or moderate the observed relationships. Fourth, no qualitative data were collected to contextualize patients' experiences of stigma or social withdrawal, which limits the depth of interpretation (40).

To address these limitations, future studies should adopt longitudinal designs to examine the temporal sequence and causal pathways linking stigma, resilience, and social alienation. Incorporating standardized measures of social support and psychiatric history would allow for more comprehensive modeling and risk adjustment. Furthermore, integrating qualitative interviews or mixed-methods designs could capture the nuanced coping strategies and contextual factors shaping psychosocial outcomes. Finally, intervention studies, particularly within community health settings, are needed to evaluate the effectiveness of resilience-building programs and stigma-reduction education in improving social reintegration and quality of life among CRC patients.

## 6 Conclusion

In summary, this study highlights the significant role of psychological resilience in mediating the relationship between perceived stigma and social alienation among postoperative CRC patients. The findings underscore the need for comprehensive care approaches that address not only the physical but also the psychosocial aspects of patient wellbeing. By integrating resilience-building interventions and stigma-reduction efforts into postoperative care, healthcare providers can enhance social reintegration and overall quality of life for CRC survivors.

## Data Availability

The raw data supporting the conclusions of this article will be made available by the authors, without undue reservation.
